# Psychological and Demographic Predictors of Insomnia Severity: Evidence from a Community Sample in Ecuador

**DOI:** 10.3390/bs15111553

**Published:** 2025-11-14

**Authors:** Daniel Oleas, Paula Alvarado-Zurita, Elías Briones, Elizabeth Terranova, Nadia Soria-Miranda, Jose A. Rodas

**Affiliations:** 1 Dirección de Investigación, Universidad Ecotec, Km. 13.5 Samborondón, Samborondón EC092302, Ecuador; daoleas@ecotec.edu.ec; 2 Escuela de Psicología, Universidad Espíritu Santo, Samborondón EC092301, Ecuador; 3 Facultad de Ciencias Psicológicas, Universidad de Guayaquil, Guayaquil EC090510, Ecuadornadia.soriam@ug.edu.ec (N.S.-M.); 4 School of Psychology, University College Dublin, D04 V1W8 Dublin 4, Ireland

**Keywords:** insomnia, perceived stress, generalized anxiety, sociodemographic factors, Ecuador, sleep health

## Abstract

(1) Background: Insomnia is one of the most prevalent sleep disorders worldwide and a growing public health concern, yet the combined contribution of psychological and demographic factors remains understudied in Latin America. This study aimed to examine how perceived stress, generalized anxiety, and sociodemographic variables predict insomnia severity in Ecuadorian adults. (2) Methods: A community sample of 698 participants (31.7% men; M = 25.6 years, SD = 10.4) completed an online survey during nationwide electricity blackouts in 2024. Measures included the Insomnia Severity Index (ISI), the Perceived Stress Scale (PSS-10), the Generalized Anxiety Disorder Scale (GAD-7), and a demographic questionnaire. Hierarchical linear regressions were conducted to evaluate the relative contribution of predictors. (3) Results: Demographic variables (age, sex, employment status, and socioeconomic level) were not significant predictors of insomnia severity (R^2^ = 0.007). Perceived stress strongly predicted insomnia (ΔR^2^ = 0.207, *p* < 0.001), and generalized anxiety added further explanatory power (ΔR^2^ = 0.074, *p* < 0.001), with both factors contributing independently (β ≈ 1.96 and β ≈ 2.67). Interaction effects with socioeconomic status were non-significant. (4) Conclusions: Psychological factors explained nearly one-third of the variance in insomnia severity, underscoring the importance of stress and anxiety as core predictors and supporting the need for integrated psychological interventions to improve sleep health in Ecuador.

## 1. Introduction

Sleep is a fundamental biological function that supports both physical and psychological health, occupying nearly one-third of the human lifespan (eBioMedicine, 2023). It plays a central role in maintaining homeostasis by supporting processes such as memory consolidation, immune functioning, hormonal regulation, and tissue repair (Zielinski et al., 2016). Adequate sleep is therefore essential for daytime performance and nighttime restoration (Scott et al., 2021). Conversely, when sleep is insufficient or of poor quality, it can act as a precipitating or aggravating factor for a wide range of health conditions, including cardiovascular disease (Diamond & Ismail, 2021), type 2 diabetes (Diamond & Ismail, 2021), metabolic syndrome (Lian et al., 2019), psychiatric disorders (Lian et al., 2019), and even certain cancers (Ruan et al., 2023; Cooper et al., 2025).

Among the various sleep disorders, insomnia is one of the most common and burdensome. It is characterized by persistent difficulties in initiating or maintaining sleep, as well as non-restorative rest (Morin & Benca, 2012; Roth, 2007). In the United States, prevalence estimates range from 16% to 22% when transient or short-term symptoms are included (Benjafield et al., 2024), and rates can reach up to 35% among older adults (Mookerjee et al., 2023). Despite advances in pharmacological and behavioral treatments, many patients experience relapse or resistance to intervention (Klugherz et al., 2023). These clinical challenges are partly explained by the complex psychoneurobiological basis of insomnia (He et al., 2024), in which physiological hyperarousal and dysregulation of the stress system have been identified as key mechanisms (Riemann et al., 2023, 2025).

Insomnia has also been consistently associated with a wide range of sociodemographic and psychological factors. Women and older adults show higher prevalence rates (Chapman et al., 2025), as do individuals facing socioeconomic disadvantage or unemployment (Etindele Sosso et al., 2023). Psychological factors such as perceived stress, anxiety, and depression further exacerbate insomnia symptoms and are known to interact with sociodemographic characteristics, amplifying vulnerability to sleep disturbances (Palagini et al., 2022). These interactions are clinically meaningful, as stress and anxiety not only increase symptom severity but also hinder treatment response and elevate relapse rates (Riemann et al., 2022). Environmental conditions, including noise, light exposure, and extreme temperatures, may further contribute to insomnia onset or maintenance.

In Latin America, insomnia affects an estimated 15% to 35% of adults, with higher rates observed among women, older individuals, and those with chronic medical conditions (Etindele Sosso et al., 2023). It is frequently associated with comorbidities such as depression, hypertension, and cardiovascular disease, leading to a marked deterioration in quality of life (Babicki et al., 2023). Beyond its clinical impact, insomnia also poses a substantial socioeconomic burden, reducing work productivity and increasing healthcare expenditures (Chaput et al., 2023; Valladares-Garrido et al., 2025).

Evidence from Ecuador reflects similar trends. In rural communities, the proportion of individuals reporting poor sleep quality increased from 29% to 49% after the COVID-19 pandemic, with higher risk among seropositive participants (Del Brutto et al., 2021). Poor sleep quality has also been identified as a predictor of increased mortality over six years of follow-up (Del Brutto et al., 2024) and is associated with greater arterial stiffness, suggesting cardiovascular consequences (Del Brutto et al., 2019). Among university students, excessive daytime sleepiness has been linked to poor sleep quality and academic stress (Rodriguez et al., 2021). Collectively, these findings show that sleep problems in Ecuador are not limited to subjective discomfort but extend to metabolic, cardiovascular, and behavioral health outcomes.

Despite these insights, no studies in Ecuador have systematically examined the combined contribution of psychological and demographic factors to insomnia. This gap limits understanding of how stress and anxiety interact with variables such as age, sex, employment status, and socioeconomic level in shaping sleep problems. Given that these interactions may reveal whether the impact of psychological distress varies across sociodemographic groups, assessing them provides a more nuanced view of vulnerability to insomnia. Therefore, the present study aimed to evaluate the relative contribution of perceived stress and generalized anxiety to insomnia severity, while accounting for demographic variables and testing potential moderating effects of socioeconomic status, in a community sample of adults from Guayaquil.

## 2. Materials and Methods

### 2.1. Participants

A total of 698 adults residing in Ecuador participated in the study (31.7% men; M = 25.6, SD = 10.4). Most participants were university students and from lower-middle socioeconomic backgrounds. Detailed demographic characteristics are presented in Table 1.

Participation was voluntary and no compensation was offered. Inclusion criteria were (a) being 18 years of age or older, and (b) residing in Ecuador at the time of the study. Cases with extensive missing data or duplicate responses were excluded. Participants with minimal missing values (<20%) were retained, and these values were later handled through multiple imputation procedures.

### 2.2. Instruments

#### 2.2.1. Demographic Questionnaire

A short ad hoc form was used to collect information about age, sex, education, marital status, employment, and socioeconomic status.

#### 2.2.2. Generalized Anxiety Disorder Scale (GAD-7, Spitzer et al., 2006)

This self-report instrument consists of seven items rated from 0 (not at all) to 3 (nearly every day), assessing symptoms of generalized anxiety according to DSM-5 criteria. The Spanish version has been culturally adapted and validated for Spanish-speaking populations (Garcia-Campayo et al., 2010) and has also demonstrated solid psychometric properties in Ecuadorian university samples (Moreno-Montero et al., 2024). Internal consistency in the current study was high (α = 0.91).

#### 2.2.3. Perceived Stress Scale (PSS-10, Cohen et al., 1983)

This widely used 10-item self-report measure assesses perceived stress over the past month. Items are rated on a Likert scale from 0 (never) to 4 (very often), with higher scores indicating greater perceived stress. The Ecuadorian adaptation has demonstrated adequate psychometric properties (Ruisoto et al., 2020). In the present study, internal consistency was good (Cronbach’s α = 0.85).

#### 2.2.4. Insomnia Severity Index (ISI, Bastien, 2001)

The ISI is a seven-item self-report tool that evaluates nighttime difficulties, dissatisfaction with sleep, and daytime consequences of insomnia. Items are scored from 0 (not at all) to 4 (very severe). Total scores are categorized as follows: 0–7 = no clinical insomnia; 8–14 = subclinical insomnia; 15–21 = moderate insomnia; 22–28 = severe insomnia. The Spanish version has shown adequate psychometric properties (Fernandez-Mendoza et al., 2012). Internal consistency in this sample was acceptable (α = 0.80).

### 2.3. Procedure

Data were collected through an online survey distributed via social media (Facebook, Instagram, and WhatsApp) and institutional mailing lists over a four-week period between October and November 2024. Data collection coincided with nationwide electricity blackouts in Ecuador, during which citizens experienced scheduled power cuts lasting up to eight hours per day. This overlap was unplanned, and the study was not designed to examine the effects of this situation. On the first page of the online form, participants were presented with a detailed informed consent statement describing the study’s purpose, voluntary participation, confidentiality guarantees, and the right to withdraw at any time without penalty. Only those who provided consent could access the questionnaire, thereby giving digital informed consent.

The survey included a short demographic section followed by the PSS-10, GAD-7, and ISI (in that order). Completion time was approximately 15–20 min, and participants could submit only one response. Duplicate entries and inconsistent or incomplete responses were removed. All participants were adults residing in Ecuador. The study complied with the Declaration of Helsinki and was approved by the Research Committee of the Psychology Program at Universidad ECOTEC.

### 2.4. Data Analysis

Analyses were conducted using R (version 4.5.1, R Core Team, 2025). First, descriptive statistics and distributional checks were performed for the main variables.

Then, a series of hierarchical linear regressions were then conducted with insomnia severity (ISI total score) as the dependent variable:

- Model 1: Demographic variables (age, sex, employment status, socioeconomic status). SES was converted to a numeric variable to allow its inclusion in correlation and regression models.
- Model 2: Addition of perceived stress (PSS-10).
- Model 3: Addition of generalized anxiety (GAD-7).
- Model 4: Inclusion of interaction terms between SES and both PSS-10 and GAD-7.

Incremental variance explained (ΔR^2^) was reported at each step and missing values for regressions were addressed using multiple imputation (20 imputed datasets), a procedure that reduces bias and preserves statistical power compared to complete-case analysis (Beesley et al., 2021).

## 3. Results

### 3.1. Insomnia Severity Distribution

**Figure 1:**
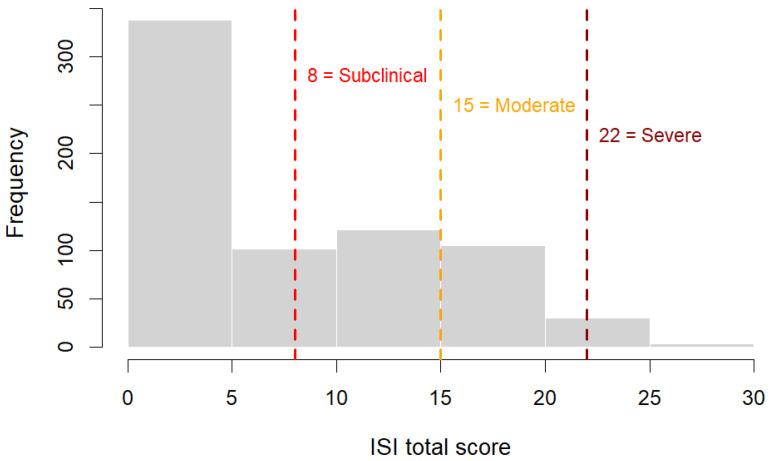
Distribution of Insomnia Severity Index (ISI) scores with clinical cut-off points (8, 15, 22) indicating subclinical, moderate, and severe insomnia categories.

Based on ISI clinical thresholds, 53.3% of participants scored within the non-clinical range (0–7), 23.5% in the subclinical range (8–14), 20.6% in the moderate range (15–21), and 2.6% in the severe range (22–28). This indicates that nearly one in four individuals presented subclinical insomnia symptoms, while a smaller proportion showed clinically significant insomnia severity. Figure 1 shows the distribution of ISI scores across the four clinical categories. The majority of participants reported no clinical insomnia, with progressively fewer cases in the subclinical, moderate, and severe ranges.

### 3.2. Descriptive and Correlation

Descriptive statistics and bivariate correlations with Spearman coefficient for the main variables are presented in Table 2. Insomnia severity was moderately correlated with both perceived stress (ρ = 0.47, *p* < 0.001) and anxiety (ρ = 0.49, *p* < 0.001). Stress and anxiety were strongly correlated (ρ = 0.64, *p* < 0.001). Age showed a small negative correlation with stress (ρ = −0.22, *p* < 0.001) but was not significantly associated with anxiety or insomnia.

### 3.3. Hierarchical Regression

Hierarchical regression analyses were conducted to examine the relative contribution of demographic and psychological variables to insomnia severity (Table 3). In Model 1, which included only demographic covariates (age, sex, employment status, and socioeconomic level), none of the predictors reached statistical significance, and the explained variance was minimal (R^2^ = 0.007). When perceived stress (PSS-10) was added in Model 2, the model showed a substantial improvement (ΔR^2^ = 0.207, *p* < 0.001), with higher stress scores significantly predicting greater insomnia severity. Model 3 further incorporated generalized anxiety (GAD-7), which also emerged as a significant predictor alongside perceived stress, increasing the explained variance to R^2^ = 0.288 (ΔR^2^ = 0.074, *p* < 0.001). Finally, Model 4 included interaction terms between socioeconomic status and both psychological predictors; these interactions were non-significant, and the overall increase in variance explained was negligible (ΔR^2^ = 0.002). Thus, psychological factors—particularly stress and anxiety—accounted for the largest share of variance in insomnia severity, whereas demographic variables and their interactions contributed little to the models.

## 4. Discussion

The present study examined how perceived stress, generalized anxiety, and demographic factors contribute to insomnia severity in Ecuadorian adults. Consistent with our hypotheses, psychological factors emerged as the strongest predictors of insomnia, whereas demographic variables showed little explanatory power. Together, stress and anxiety accounted for nearly one-third of the variance in insomnia severity, confirming their central role in the experience of sleep difficulties. In contrast, age, sex, employment status, and socioeconomic level were not significant predictors. The absence of significant interactions between psychological and sociodemographic variables further indicated that the impact of stress and anxiety on sleep was similar across demographic groups.

These findings emphasize that insomnia is primarily influenced by psychological rather than sociodemographic mechanisms. In particular, perceived stress and anxious arousal appear to act as proximal determinants of sleep disturbance, consistent with psychobiological models of hyperarousal that describe insomnia as a disorder of sustained cognitive and physiological activation (Kalmbach et al., 2018; Riemann et al., 2023). The progressive increase in explained variance across the hierarchical models (ΔR^2^ = 0.207 with stress and ΔR^2^ = 0.074 with anxiety) underscores the robustness of these psychological predictors. The results also support the conceptualization of insomnia as a transdiagnostic condition, sharing emotional and cognitive mechanisms with other stress-related and anxiety disorders, and point to the value of integrated approaches that address these overlapping processes.

The strong association between perceived stress and insomnia severity aligns with previous studies showing that higher stress levels predict sleep disruption across diverse populations, including students, healthcare workers, and patients with chronic illness (Tao et al., 2023; Carrasco et al., 2024; Strete et al., 2025). In the Ecuadorian context, widespread social instability and the 2024 electricity blackouts may have amplified perceptions of uncertainty and loss of control, reinforcing stress-related sleep problems. This context likely increased the salience of stress as a determinant of insomnia. The present findings thus contribute regional evidence to a consistent global pattern: chronic stress undermines sleep regulation through heightened cognitive arousal and emotional tension.

Anxiety also showed an independent contribution to insomnia, even after controlling for stress. This supports the idea that both constructs, though correlated, capture distinct pathways to sleep disruption. Anxiety’s effect may operate through excessive worry and rumination, which prolong nocturnal wakefulness and impair sleep onset (Cox & Olatunji, 2016). The coexistence of stress and anxiety as significant predictors suggests that insomnia severity is best explained by the joint action of these psychological factors. These results highlight the need for interventions that simultaneously target stress management and anxiety reduction, as addressing only one component may be insufficient to improve sleep outcomes.

Although demographic variables were included in the analysis, none showed significant predictive power once psychological factors were considered. This finding suggests that demographic characteristics such as sex, age, employment status, and socioeconomic level play a limited role when stress and anxiety are taken into account. Previous studies have identified women and older adults as being at greater risk for insomnia (B. Kim et al., 2022; Y. Kim et al., 2025; Kiss et al., 2025); however, the present results indicate that these differences may be mediated by psychological processes rather than demographic attributes themselves. For example, women’s higher vulnerability to insomnia may be better explained by elevated stress sensitivity and difficulties in emotion regulation rather than by biological sex per se (Yu et al., 2025). The predominance of young female participants in our sample may also have reduced the variability necessary to detect demographic effects.

The lack of association between age and insomnia in this study is also consistent with the sample profile and contextual conditions. Most participants were young adults (M = 25.6), a stage of life characterized by high exposure to academic and occupational stressors that may overshadow age-related differences (Cañarte-Murillo & Parrales-Figueroa, 2024). In addition, data collection took place during a period of nationwide power outages, which likely acted as a shared environmental stressor across all age groups, minimizing demographic variability. These results highlight how acute societal conditions can temporarily homogenize psychological experiences such as stress and sleep disturbance.

The non-significant interaction effects between psychological and socioeconomic variables further suggest that the influence of stress and anxiety on insomnia operates similarly across income levels. In other words, the psychological burden of feeling overwhelmed or anxious affects individuals regardless of their socioeconomic background. This finding aligns with recent evidence showing that stress-related hyperarousal is a universal mechanism contributing to insomnia (Riemann et al., 2025). From a public health perspective, these results reinforce the need for accessible mental health and sleep interventions that are not limited to any specific socioeconomic group. Programs that promote emotional regulation and stress management could benefit diverse populations, particularly in contexts like Ecuador, where structural and environmental stressors are common.

It is also important to consider the characteristics of the sample when interpreting these findings. Although the predominance of university students may appear as a limitation, it provides valuable information about a population segment highly exposed to psychosocial and academic demands. Previous studies have shown that young adults in higher education often experience elevated levels of stress, uncertainty about employment, and financial instability, all of which increase vulnerability to insomnia (Dinis & Bragança, 2018; Mbous et al., 2022). In this sense, the sample composition offers insight into how stress and anxiety mechanisms operate in lower and lower-middle socioeconomic contexts typical of emerging economies such as Ecuador. Nevertheless, future research should include more heterogeneous samples encompassing older and fully employed adults to examine whether the observed relationships hold across different demographic and occupational groups.

The context in which the data were collected may have played a role in the strength of the psychological associations observed. The study coincided with a period of nationwide electricity blackouts and growing social instability, conditions that may have temporarily elevated stress and anxiety levels. Although this situation was not a planned focus of the research, it likely intensified perceptions of uncertainty and lack of control, two factors strongly associated with sleep difficulties. Nevertheless, the consistency of the results with findings from other settings suggests that these associations reflect stable psychological mechanisms rather than transient reactions to a specific environmental event. Future studies could incorporate measures of situational exposure or perceived impact to better distinguish acute from chronic stress effects.

Despite the robustness of the findings, several limitations should be acknowledged. First, the cross-sectional design does not allow for conclusions about causality between psychological factors and insomnia severity. Longitudinal or experimental designs are needed to clarify whether stress and anxiety precede or result from sleep problems. Second, all variables were assessed through self-report measures, which may introduce recall or social desirability bias. Incorporating objective sleep measures such as actigraphy or polysomnography would strengthen future studies. Third, the sample was predominantly composed of young adults and women, which limits generalizability to older or more gender-balanced populations. Broader sampling strategies are recommended to validate these results across diverse age, occupational, and socioeconomic groups.

Finally, while online data collection facilitated broad participation during a period of national disruption, it also restricted access to individuals with internet connectivity and digital literacy, potentially excluding less connected or rural populations. This limitation, however, also reflects a demographic reality of modern Ecuador, where young, urban, digitally active adults represent the most reachable population segment for mental health surveys. Future research could address this limitation by combining online and field-based data collection to ensure broader representativeness.

## 5. Conclusions

This study highlights the pivotal role of psychological factors—particularly perceived stress and generalized anxiety—in predicting insomnia severity, while demographic variables showed minimal explanatory power. Stress and anxiety contributed uniquely and jointly, accounting for nearly one-third of the variance, and their effects were consistent across socioeconomic strata. These findings reinforce the conceptualization of insomnia as a transdiagnostic condition rooted in cognitive and emotional hyperarousal, underscoring the need for early detection and integrated psychological interventions that target both stress and anxiety. In the Ecuadorian context, where social and environmental stressors are prevalent, such approaches may be especially relevant to improve sleep health and prevent associated mental and physical health consequences.

## Figures and Tables

**Table 1:** Demographic characteristics of the sample (N = 698).

Variable	Category	n	% ^1^
Sex	Male	221	31.7
	Female	477	68.3
Employment status	Employed	163	23.4
	Self-employed	57	8.2
	Unemployed	91	13.0
	Student	351	50.3
	Business owner	22	3.2
	NA	14	2.0
Socioeconomic level	Low	78	11.2
	Lower-middle	445	63.8
	Upper-middle	171	24.5
	High	2	0.3
	NA	2	0.3

^1^ Percentages are based on valid cases.

**Table 2:** Descriptive analysis and Correlation.

Variable	M	SD	Min	Max	1	2	3	4
1. Age	25.57	10.40	16	68	–			
2. SES	1.14	0.59	0	3	0.00	–		
3. Anxiety (GAD-7)	7.75	5.19	0	21	−0.07	−0.05	–	
4. Insomnia severity (ISI)	7.39	7.66	0	28	−0.04	−0.19 *	0.49 **	–
5. Perceived stress (PSS-10)	18.83	6.11	0	38	−0.22 **	−0.03	0.64 **	0.47 **

* = *p* < 0.05, ** = *p* < 0.001.

**Table 3:** Hierarchical regression analyses for insomnia.

Predictors	M1 β [95% CI]	M2 β [95% CI]	M3 β [95% CI]	M4 β [95% CI]
Age	−0.05 [−0.67, 0.56]	0.59 * [0.03, 1.14]	0.41 [−0.12, 0.94]	0.42 [−0.11, 0.96]
Sex (Female)	0.86 [−0.37, 2.09]	−0.66 [−1.78, 0.46]	−0.83 [−1.90, 0.24]	−0.82 [−1.89, 0.25]
Employment status	0.35 [−0.13, 0.83]	0.17 [−0.26, 0.60]	0.20 [−0.21, 0.61]	0.21 [−0.20, 0.62]
Socioeconomic status	0.05 [−0.52, 0.62]	0.35 [−0.16, 0.86]	0.25 [−0.24, 0.74]	0.27 [−0.22, 0.76]
Perceived Stress (PSS-10)	—	3.65 ** [3.12, 4.18]	1.96 ** [1.32, 2.60]	1.95 ** [1.30, 2.59]
Generalized Anxiety (GAD-7)	—	—	2.67 ** [2.05, 3.29]	2.67 ** [2.05, 3.30]
PSS-10 × Socioeconomic status	—	—	—	0.23 [−0.36, 0.83]
GAD-7 × Socioeconomic status	—	—	—	0.07 [−0.49, 0.64]
R^2^	0.007	0.214	0.288	0.289
ΔR^2^	—	0.207 **	0.074 **	0.002

Standardized coefficients (β) with 95% confidence intervals in brackets. ΔR^2^ reflects the change in explained variance compared to the previous model. * = *p* < 0.05, ** = *p* < 0.001.

## Data Availability

The raw data and original data presented in the study are openly available in Open Science Framework at https://osf.io/r3wmq/ (accessed on 11 September 2025).
